# Associations between age and dyslipidemia are differed by education level: The Cardiovascular and Metabolic Diseases Etiology Research Center (CMERC) cohort

**DOI:** 10.1186/s12944-020-1189-y

**Published:** 2020-01-18

**Authors:** So Mi Jemma Cho, Ho Jae Lee, Jee Seon Shim, Bo Mi Song, Hyeon Chang Kim

**Affiliations:** 1 Department of Public Health, Yonsei University Graduate School, Seoul, South Korea; 2 Department of Preventive Medicine, Yonsei University College of Medicine, 50-1 Yonsei-ro, Seodaemun-gu, Seoul, 03722 Republic of Korea; 3 Cardiovascular and Metabolic Diseases Etiology Research Center, Yonsei University College of Medicine, Seoul, South Korea

**Keywords:** Dyslipidemias, Aging, Education, Risk factors

## Abstract

**Background:**

Dyslipidemia is a multifactorial disorder, which arises from complex interactions among genetic and environmental risk factors. Previous studies have established the deteriorating effect of aging on lipid profiles. However, little is known about the role of education level, a stable marker of socioeconomic status, which reflect modifiability of lifestyle risk factors. Therefore, we examined the association between age and individual dyslipidemia parameter across education level among healthy, middle-aged Korean women.

**Methods:**

From 2049 middle-aged women, education attainment was classified into completion of elementary school or below, middle school, high school, college or above. Dyslipidemia was assessed in adherence to the 2018 Korean Dyslipidemia Treatment Guideline. Multivariable logistic regression and generalized linear model tested for associations between age and dyslipidemia parameter across education level and other known risk factors, including menopause, obesity, and current drinking and smoking.

**Results:**

In this cross-sectional analysis, the prevalence of each dyslipidemia parameter was significantly different by age and education level. The odds ratio (OR) for dyslipidemia was higher among participants who were older and had received higher education (OR = 2.31, *p* for interaction = 0.008) than younger and low education counterpart. The interaction between age and education level remained significant for hypercholesterolemia (*p* for interaction = 0.003) and hyper-LDL-cholesterolemia (*p* for interaction = 0.002).

**Conclusions:**

Separate examination of individual dyslipidemia parameter indicated varying degree of interaction with age and education level. Such results imply that each type of lipid abnormality may arise from and be exacerbated by heterogeneous composition of biological and lifestyle risk factors, which may be reflected by education level.

## Introduction

Dyslipidemia is a multi-etiologic and polygenic disorder that arises from complex interactions among genetic, environmental, behavioral, and social risk factors [1, 2]. Previous studies have established that adverse lipid levels independently contribute to development and progression of atherosclerotic cardiovascular and coronary heart diseases (ASCVD and CHD) [3–5]. Lipid abnormality is particularly concerning in women, as they undergo drastic escalation with biological aging and menopause-related endocrine changes, triumphing those of men by the fifth decade [6]. In 2017, the Global Burden of Disease study reported that high concentrations of cholesterols caused about 4.4 million deaths and 93.8 million disability-adjusted life years, representing the seventh leading risk factors globally for women [7, 8]. Analogously, the recent Korean statistics pinpointed age-dependent drastic increase in dyslipidemia prevalence in women, mounting from 14.9% in third decade to 56.4% by the age of 60 [9].

Along with biological age, socioeconomic status (SES) is a pronounced risk factor of dyslipidemia, that is implicated with modifiable lifestyle risk factors, such as cigarette smoking, alcohol consumption, and physical inactivity. Moreover, SES is also associated with differential exposure to physiological and psychological stress and material resources, including timely access to adequate health care [10]. In particular, education level is one of the most commonly utilized markers of SES that is relatively stabilized in early periods of life, unlike income or occupation. It is resistant to changes in life-course circumstances or health [11]. It is suggested that education enables people to integrate healthy behaviors into a coherent lifestyle, thereby affecting health-related choices, independent of parental, spousal, or neighborhood SES status [12].

Previous literature regarding the role of education on dyslipidemia have been inconsistent by population characteristics. In a Korean study that examined association between the SES and dyslipidemia, the risks for hypoalphalipoprotein and hypertriglyceridemia increased steadily with decreasing household income and education level [13]. However, in a multinational study assessing the association of lifelong education level with subclinical atherosclerosis, the results were defined in men only [14]. With accumulated evidence suggesting the disproportionate risk associated with a broad range of unhealthy lifestyle factors manifested by education level, it is crucial to examine the role of education on risk of dyslipidemia.

The prevalence of individual parameter of dyslipidemia and its association with age and education level have not been fully investigated in Korean population. Hitherto, the objective of this study was to describe age-specific prevalence rates of individual parameter of dyslipidemia among community-dwelling middle-aged Korean women, a sex demonstrating more heterogeneous education background. Then, we identified whether there are significant interactions observed on dyslipidemia prevalence between age and education level. We hypothesized that the association between age and each dyslipidemia parameter will be differed by education level in different directions and magnitudes.

## Methods

### Study population

The study participants consisted of female, community-dwelling, capital residents of Republic of Korea, who were enrolled in the Cardiovascular and Metabolic Diseases Etiology Research Center (CMERC) cohort. Briefly, the CMERC study aimed to identify novel risk factors and to investigate distribution and effects of known cardiac and metabolic diseases risk factors, ultimately to develop improved cardiovascular disease prediction tools for the general Korean population [15]. Using validated questionnaire, the trained interviewee collected detailed information on SES, health behaviors, disease history, nutrition, and psychosocial characteristics. Adhering to standardized protocols, anthropometry and blood and urinal profiles were also assayed to identify high-risk individuals who will merit from earlier intervention [15]. The exact details of the CMERC study has been published elsewhere [15]. In the present study, among 3332 participants who have undergone baseline examination between 2013 and 2017, participants with history of malignant cancer, overt cardiovascular diseases, or missing information on lipid profiles glycemic index were excluded, yielding 2049 participants for the final analysis.

This study has been approved by the institutional review boards of Severance Hospital, Yonsei University Health System, Seoul, Korea (4–2013-0661). Written informed consent has been obtained from all participants prior to the baseline survey. Participants were ensured that they can withdraw from the study at any time, regardless of its cause.

### Questionnaire survey and health examination

A face-to-face interview obtained details on the following demographic characteristics and health-related behaviors. Age was cross-referenced with government-issued identification and obtained in years. Then, we divided age into four groups: 30–39 years, 40–49 years, 50–59 years, and 60–64 years and, again, by median age of 54 years. Household income was obtained in the nearest Korean won then categorized into cohort-specific quartile. Current occupation was classified into white and blue collar or unemployed. Education level was categorized into completion of elementary school or below, middle-school, high school, or college/university; it was also categorized into low (completion of high school or below) and high (college degree or above). Physical activity was assessed by the Korean version of the International Physical Activity Questionnaire (IPAQ) standard [16] to yield metabolic equivalent of task (MET). Alcohol consumption was recorded as the average frequency and amount of intake over the past year, separately by the type of alcoholic beverage. Cigarette smoking status was recorded as average packs per day, likewise over the past year. Information on reproductive health included menopausal status, defined by cessation of menstruation for a minimum of one consecutive year, number of and pregnancy and its successive outcomes (childbirth, miscarriage, abortion, and still-birth), history of gestational hypertension or diabetes, and oral contraceptive/hormonal replacement therapy usage duration. Dietary patterns were evaluated using a semi-quantitative food frequency questionnaire, which was developed and validated for the general Korean population [17]. Major macro- and micro-nutrients, such as daily caloric, carbohydrates, fat, and sodium intake, were calculated. Information on familial and personal morbidity history included the age at the first diagnosis of hypertension, diabetes mellitus, fatty liver, and so on. Accordingly, the participants presented prescription record entailing previous and current treatment status, including lipid-lowering medications prescribed. The quality of the survey was controlled by trained personnel using calibrated equipment and strict adherence to standardized protocols.

### Anthropometric measurements and biochemical tests

Height was measured to the nearest 0.1 cm using stadiometers: a DS-102 (Jenix, Seoul, Korea), and weight was measured to the nearest 0.1 kg on a digital scale: a DB-150 (CAS, Seongnam, Korea). To minimize measurement variability, a zero-point adjustment was conducted at least once a week using a standard ruler (170 cm) and weights (20, 40, and 60 kg). Body mass index (BMI) was, then, calculated as a ratio of weight in kilograms to height in squared meters [18]. Blood pressure was measured using both single- and double-arm automated oscillometric device (HEM-7080, Omron Health, Matsusaka, Japan and HEM-9000 AI, Omron Health). Overnight-fasting blood samples and casual urine samples were obtained in the morning, and bioassays were performed at a single laboratory (Seoul Clinical Laboratories R&D Center, Seoul, Korea). Serum lipid markers, including total cholesterol (TC), triglycerides (TG), high-density lipoprotein cholesterol (HDLC), and low-density lipoprotein cholesterol (LDLC) levels were analyzed enzymatically with an ADIVA 1800 AutoAnalyzer (Siemens Medical Sol.).

### Definition of dyslipidemia

From eight-hours fasting serum, total cholesterol (TC), triglycerides (TG), high-density lipoprotein cholesterol (HDLC) and low-density lipoprotein cholesterol (LDLC) levels were analyzed enzymatically with an ADVIA 1800 AutoAnalyzer (Siemens Medical Sol.). In this analysis, we presented the distribution of TG in its logarithmic form due to skewed distribution. Dyslipidemia was defined based on the 2018 Korean Dyslipidemia Treatment Guideline [19], which is equivalent to the Adult Treatment Panel III guidelines [20]. Hypercholesterolemia was defined as TC ≥240 mg/dL; hypertriglyceridemia was defined as TG ≥200 mg/dL; hypoalphalipoproteinemia was defined as HDLC < 40 mg/dL; hyper-LDL-cholesterolemia was defined as LDLC ≥160 mg/dL. Having any one type of the aforementioned cholesterol abnormality or current intake of lipid-lowering agent was regarded as prevalent dyslipidemia.

### Statistical analyses

General characteristics of the study population were reported as frequency and percentage or mean and standard deviation. Then, they were compared via independent t-test, the Wilcoxon rank sum test, chi-square test for differences, or analysis of variance test for multiple comparisons. Prevalence of dyslipidemia and its parameter were calculated separately by age and education level. We used multivariable logistic regression to calculate odds ratio (OR) and 95% confidence interval (CI) to calculate the risk associated with older age across education level. Then, we employed generalized linear model to identify presence of interaction between age and education level and other known risk factors of dyslipidemia. Here, we tested for interaction between age (both as continuous and categorical) and education (both as aforementioned four categories and binary by higher education). The final model was adjusted for BMI, reproductive history, household income, occupation, education level, current drinking and smoking status, physical activity, and current intake of lipid-lowering agents. Hosmer-Lemeshow goodness of fit for logistic regression and C-statistic ensured appropriateness of the model. Sensitivity analyses were conducted by using lower LDLC cutoffs in the context of secondary prevention. Specifically, we referred to the 2018 Korean dyslipidemia guidelines [19] and the 2019 European Society of Cardiology/European Atherosclerosis Society guidelines for the management of dyslipidemia’s^21^ target LDLC ≥130 mg/dL for persons with low ASCVD risk and LDLC ≥116 mg/dL for moderate ASCVD risk. Those within these elevated LDLC ranges are recommended lifestyle modification or/and initiation/intensification of pharmacological treatment. All statistical tests were two-sided, and the statistical significance was set at a *p*-value< 0.05. All analyses were performed using SAS version 9.4 (SAS Institute Inc., Cary, NC).

## Results

### Participant characteristics

A total of 821 out of 2049 female participants (40.1%) had dyslipidemia (Additional file 1: Fig. S1). Table 1 presents the general characteristics of the study participants by age group and completion of higher education. When stratified by decile age group, the oldest group had the lowest proportion of higher education, household income, employment, current smoking and drinking, and average caloric intake. Yet, women in their fifth decade presented the highest TC, TG, and LDLC levels whilst the lowest HDLC level. Generally, adverse lipid profiles were accompanied by the highest percentage of obesity, hypertension, and diabetes comorbidities.

**Table 1:** General characteristics of the study population by age group and education level (*n* = 2049)

Variables	Age, year												*p-*value	Education level						*p-*value
	30–39			40–49			50–59			60–64				Low			High			
	(*n* = 313)			(*n* = 366)			(*n* = 996)			(*n* = 374)				(*n* = 1245)			(*n* = 804)			
Age, year	34.9	±	2.4	45.2	±	2.9	55.2	±	2.7	61.7	±	1.8	< 0.0001	54.1	±	7.3	47.4	±	9.9	< 0.0001
Body mass index, kg/m^2^	22.1	±	3.0	23.1	±	2.9	23.6	±	2.8	24.1	±	2.9	< 0.0001	23.7	±	2.9	22.9	±	2.9	< 0.0001
Education level													< 0.0001							< 0.0001
Elementary school or below	0		(0.0)	4		(1.1)	79		(7.9)	72		(19.3)		155		(12.5)				
Middle school	2		(0.6)	13		(3.6)	144		(14.5)	73		(19.5)		232		(18.6)				
High school	78		(24.9)	167		(45.6)	478		(48.0)	135		(36.1)		858		(68.9)				
College or above	233		(74.4)	182		(49.7)	295		(29.6)	94		(25.1)					804		(100.0)	
Household income													< 0.0001							< 0.0001
Low	77		(24.6)	89		(24.3)	257		(25.8)	102		(27.3)		310		(24.9)	192		(23.9)	
Middle-low	71		(22.7)	69		(18.9)	236		(23.7)	82		(21.9)		294		(23.6)	207		(25.7)	
Middle-high	83		(26.5)	113		(30.9)	246		(24.7)	94		(25.1)		328		(26.4)	192		(23.9)	
High	82		(26.2)	95		(26.0)	257		(25.8)	96		(25.7)		313		(25.1)	213		(26.5)	
Occupation													< 0.0001							< 0.0001
White collar	156		(49.8)	130		(35.5)	202		(20.3)	35		(9.4)		174		(14.0)	353		(43.9)	
Blue collar	33		(10.5)	102		(27.9)	342		(34.3)	85		(22.7)		471		(37.8)	91		(11.3)	
Unemployed	124		(39.6)	134		(36.6)	452		(45.4)	254		(67.9)		600		(48.2)	360		(44.8)	
Current smoking status													< 0.0001							0.32
Non-smoker	262		(83.7)	332		(90.7)	960		(96.4)	359		(96.0)		1159		(93.1)	754		(93.8)	
Former smoker	28		(9.0)	14		(3.8)	18		(1.8)	12		(3.2)		42		(3.4)	30		(3.7)	
Current smoker	23		(7.4)	20		(5.5)	18		(1.8)	3		(0.8)		44		(3.5)	20		(2.5)	
Current drinking status													< 0.0001							0.33
Non-drinker	56		(17.9)	112		(30.6)	370		(37.2)	157		(42.0)		434		(34.9)	261		(32.5)	
Former drinker	19		(6.1)	7		(1.9)	31		(3.1)	9		(2.4)		37		(3.0)	29		(3.6)	
Current drinker	238		(76.0)	247		(67.5)	595		(59.7)	208		(55.6)		774		(62.2)	514		(63.9)	
Physical activity, MET-min/week	2134.7	±	3034.2	2309.5	±	3076.8	2663.3	±	3433.0	2644.0	±	2741.4	0.03	2810.1	±	3539.2	2060.1	±	2517.5	< 0.0001
Sedentary time, hours/day	6.6	±	3.6	6.1	±	3.3	5.5		2.9	5.5	±	2.8	< 0.0001	5.5	±	2.9	6.3	±	3.3	< 0.0001
Menopause													< 0.0001							< 0.0001
Yes	4		(1.3)	45		(87.7)	864		(86.8)	374		(100.0)		936		(75.2)	351		(43.7)	
No	309		(98.7)	321		(12.3)	132		(13.3)	0		(0.0)		309		(24.8)	453		(52.3)	
Energy intake, kcal (*n* = 2216)	2180.6	±	765.3	2058.1	±	720.1	1976.0	±	672.9	1956.2	±	645.6	< 0.0001	1988.5	±	668.4	2054.2	±	695.0	0.05
Total cholesterol, mg/dL	181.2	±	28.1	195.5	±	30.6	205.6	±	34.1	200.8	±	34.4	< 0.0001	200.6	±	33.8	196.9	±	33.6	0.01
Triglyceride, logarithmic	4.3	±	0.4	4.5	±	0.4	4.7	±	0.4	4.8	±	0.4	< 0.0001	4.7	±	0.4	4.5	±	0.4	< 0.0001
HDL cholesterol, mg/dL	62.8	±	14.5	62.8	±	14.2	60.9	±	14.4	58.1	±	13.5	< 0.0001	60.4	±	14.4	61.9	±	14.2	0.02
LDL cholesterol, mg/dL	101.5	±	24.8	112.7	±	26.8	121.3	±	29.9	117.1	±	30.8	< 0.0001	116.8	±	30.0	114.8	±	29.0	0.13
Lipid-lowering agent													< 0.0001							< 0.0001
Yes	0		(0.0)	12		(3.3)	135		(13.6)	103		(27.5)		186		(14.9)	64		(8.0)	
No	313		(100.0)	354		(96.7)	861		(86.5)	271		(72.5)		1059		(85.1)	740		(92.0)	
Obese, BMI ≥25 kg/m^2^													< 0.0001							< 0.0001
Yes	47		(15.0)	80		(21.9)	278		(27.9)	118		(31.6)		374		(30.0)	154		(19.2)	
No	266		(85.0)	286		(78.2)	718		(72.2)	256		(68.4)		871		(70.0)	650		(80.8)	
Hypertension													< 0.0001							< 0.0001
Yes	6		(1.9)	47		(12.8)	241		(24.2)	122		(32.6)		307		(24.7)	109		(13.5)	
No	307		(98.1)	319		(87.2)	755		(75.8)	252		(67.4)		938		(75.3)	695		(86.5)	
Diabetes mellitus													< 0.0001							< 0.0001
Yes	1		(0.3)	10		(2.7)	76		(7.6)	46		(12.3)		109		(8.8)	24		(3.0)	
No	312		(99.7)	356		(7.3)	920		(92.4)	328		(87.7)		1136		(91.2)	780		(97.0)	

Values are presented as mean ± standard deviation or number (%)

*P*-value was derived from the independent t-test, the Wilcoxon rank sum test, chi-square test, or analysis of variance test for multiple comparisons

Low education level refers to completion of high school or below; high education level refers to completion of college or above

Abbreviation: *BMI* Body mass index, *HDL* High-density lipoprotein, *LDL*, Low-density lipoprotein, *MET* Metabolic equivalent of task

Again, there were substantial differences by education attainment. Women in low education group were significantly older, had higher BMI and proportion of menopause yet lower average energy intake and higher physical activity level. In terms of SES indicators, there were marked differences in distribution of household income and current occupation; low education women reported lower household income and higher unemployment. In addition, there were pronounced differences in health behavior; high education women were less likely to be current smoker yet more likely to be current drinker. Compared to their counterpart, high education women presented significantly lower TC (196.9 vs. 200.6 mg/dL), log(TG) (4.5 vs. 4.7), and LDLC (114.8 vs. 116.8 mg/dL) yet and higher HDLC (61.9 vs. 60.4 mg/dL) levels.

### Prevalence of dyslipidemia

**Fig. 1:**
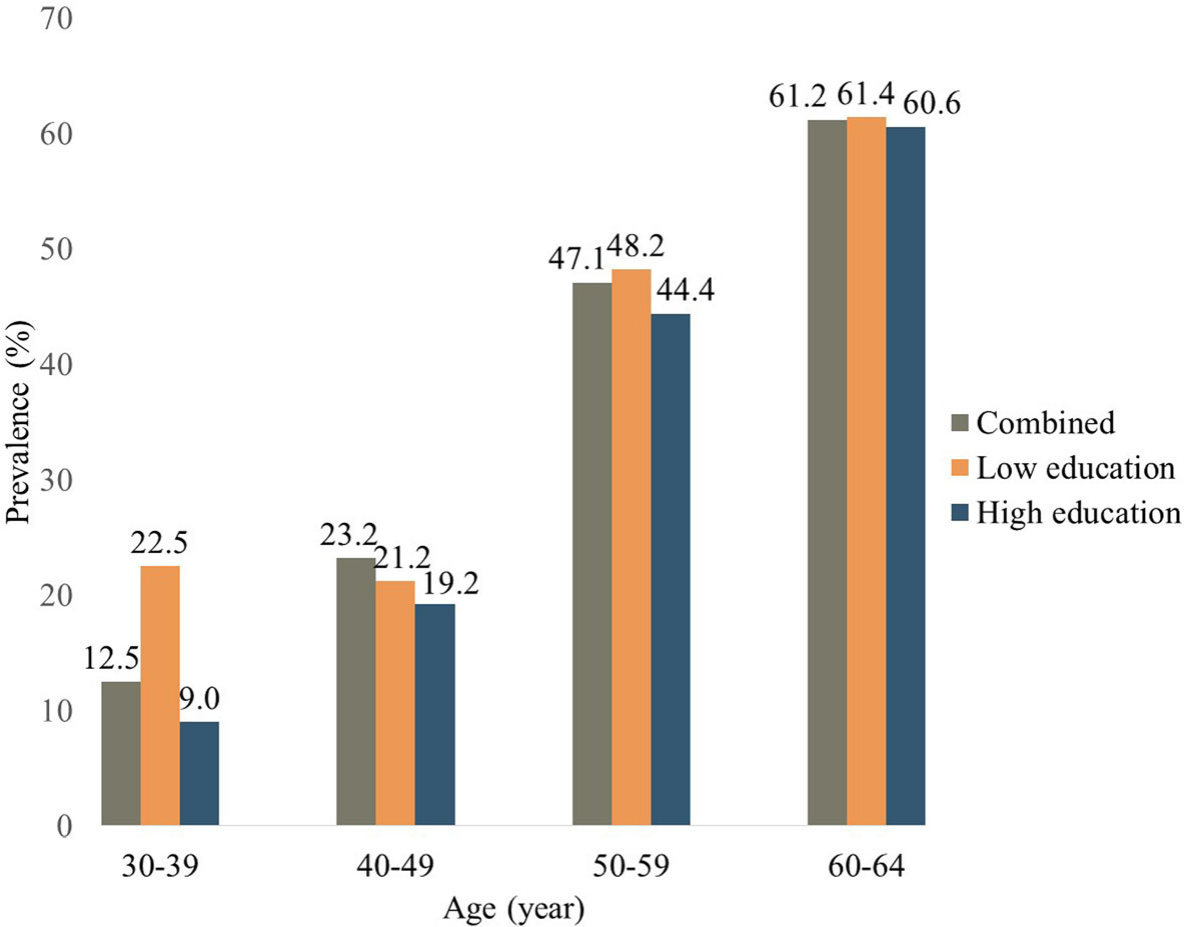
Prevalence of dyslipidemia by age group and education level (*n* = 2049). Low education level refers to completion of high school or below; high education level refers to completion of college or above

As illustrated in Fig. 1 and Table 2, the prevalence of dyslipidemia incrementally increases from the youngest group (22.5% in low education group; 9.0% in high education group) to the oldest group (61.4 and 60.6%, respectively). In all age groups, high education group had comparatively lower prevalence of dyslipidemia than the low education group.

**Table 2:** Prevalence of dyslipidemia and its parameters by age and education level (*n* = 2049)

	Age, year								^☨^*p*-value
	30–39		40–49		50–59		60–64		
Low education level									
Dyslipidemia	18	(22.5)	50	(27.2)	338	(48.2)	172	(61.4)	< 0.0001
Hypercholesterolemia	3	(3.8)	15	(8.2)	111	(15.8)	33	(11.8)	0.001
Hypertriglyceridemia	11	(13.8)	20	(10.9)	150	(21.4)	75	(26.8)	0.0004
Hypoalphalipoproteinemia	4	(5.0)	5	(2.7)	34	(4.9)	16	(5.7)	0.4512
Hyper-LDL-cholesterolemia	2	(2.5)	10	(5.4)	73	(10.4)	16	(5.7)	0.007
High education level									
Dyslipidemia	21	(9.0)	35	(19.2)	131	(44.4)	57	(60.6)	< 0.0001
Hypercholesterolemia	4	(1.7)	11	(6.0)	55	(18.6)	14	(14.9)	< 0.0001
Hypertriglyceridemia	11	(4.7)	19	(10.4)	60	(20.3)	20	(21.3)	< 0.0001
Hypoalphalipoproteinemia	5	(2.2)	3	(1.7)	12	(4.1)	5	(5.3)	0.2204
Hyper-LDL-cholesterolemia	5	(2.2)	8	(4.4)	34	(11.5)	12	(12.8)	< 0.0001

Values are presented as number (%)

^☨^*P*-value was derived from the analysis of variance test for multiple comparisons

Low education level refers to completion of high school or below; high education level refers to completion of

college or above

Abbreviation: *LDL* Low-density lipoprotein

**Fig. 2:**
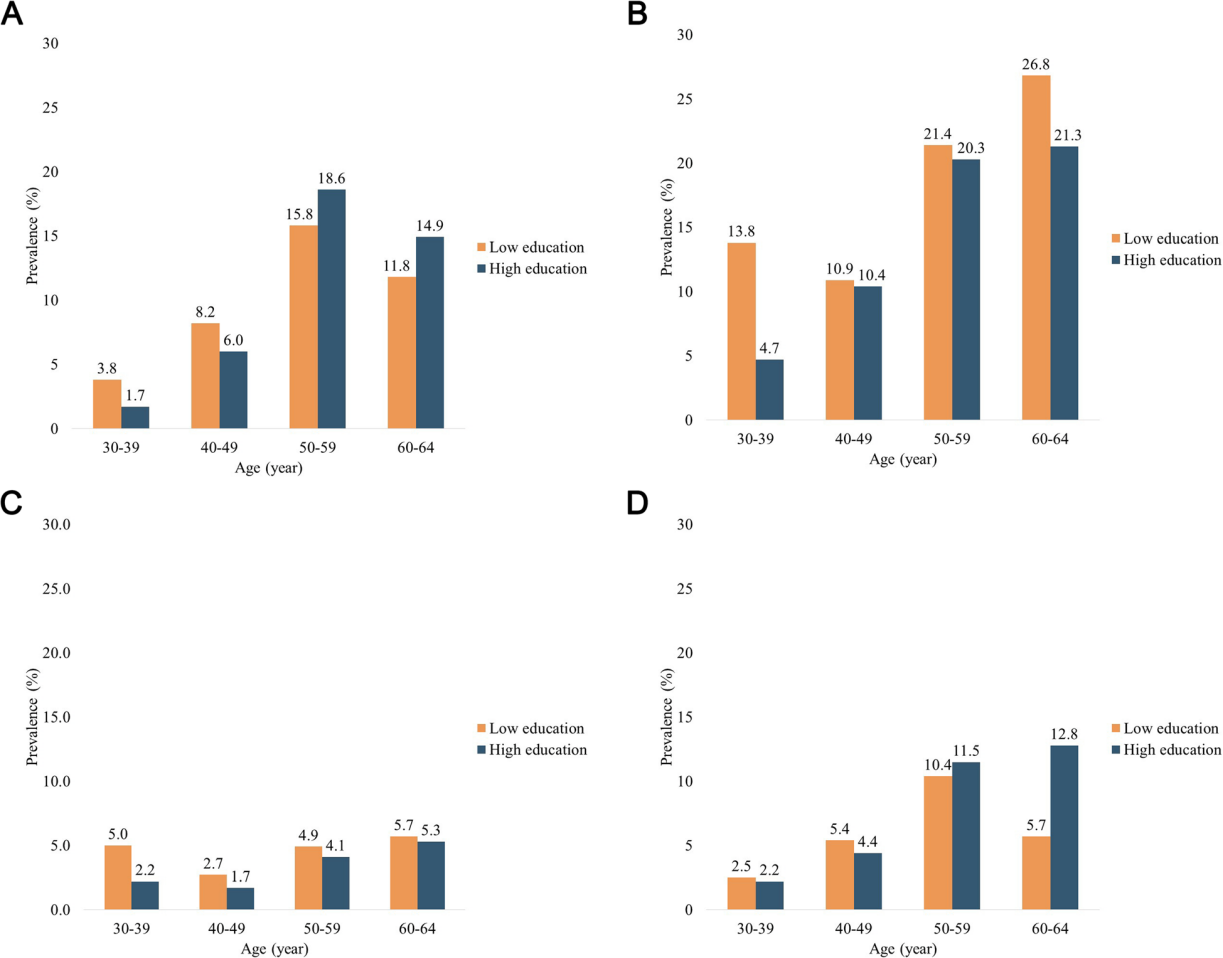
**a.** Prevalence of hypercholesterolemia by age group and education level (*n* = 2049). Low education level refers to completion of high school or below; high education level refers to completion of college or above. **b.** Prevalence of hypertriglyceridemia by age group and education level (*n* = 2049). Low education level refers to completion of high school or below; high education level refers to completion of college or above. **c.** Prevalence of hypoalphalipoproteinemia by age group and education level (*n* = 2049). Low education level refers to completion of high school or below; high education level refers to completion of college or above. **d.** Prevalence of hyper-LDL-cholesterolemia by age group and education level (*n* = 2049). Low education level refers to completion of high school or below; high education level refers to completion of college or above

However, when examining individual parameter of dyslipidemia, the age-associated trend was largely divergent by education level (Table 2 and Figs. 2). Regardless of education level, the prevalence of hypercholesterolemia acclimated to its zenith in 50–59 years group, then declined in the oldest group. Moreover, until the fifth decade, hypercholesterolemia was more common in low education group; however, its prevalence was triumphed by that of high education group after the age 50. The prevalence of hypertriglyceridemia and hypoalphaliproteinemia was consistently higher in low education group within all age groups. Interestingly, women in fourth decade had lower prevalence of hypercholesterolemia than those in third decade in both low (11.8% vs. 15.8%) and high (14.9% vs. 18.6%) education groups. Lastly, whereas the prevalence of hyper-LDL-cholesterolemia was incrementally higher with older age in high education level group, its low education counterpart showed reduction from the age 50–59 years (10.4%) to 60–64 years group (5.7%). Separate examination of each cholesterol and triglyceride levels indicated parallel results (Additional file 1: Table S1).

### Interaction between age and education level on dyslipidemia prevalence

We used multivariable logistic regression to calculate odds ratio (OR) and 95% confidence interval (CI) associated with older age across education level. Then, we employed generalized linear model to identify presence of interaction between age and education level and other known risk factors of dyslipidemia. Here, we tested for interaction between age (both as continuous and categorical) and education (both as ordinal and binary).

**Fig. 3:**
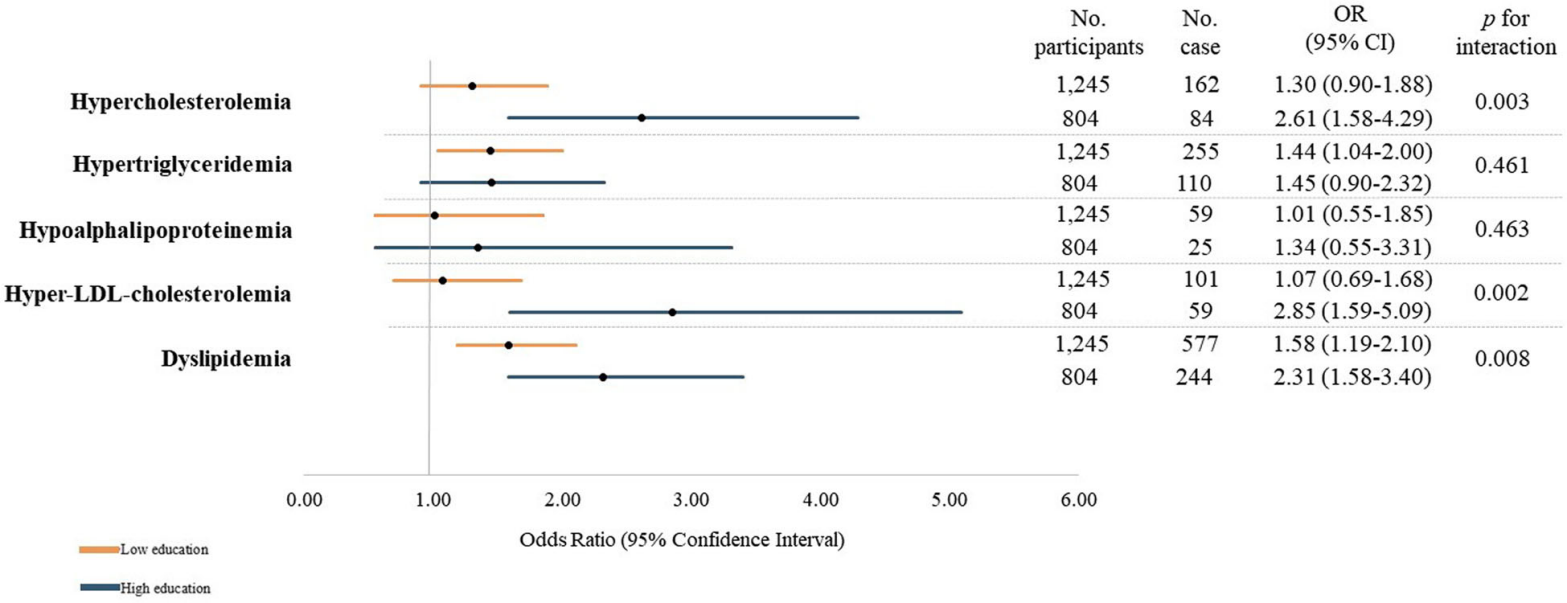
Association between and age dyslipidemia parameters according to education level using a generalized linear model (*n* = 2049). Abbreviation: LDL, low-density lipoprotein

Overall, older age was associated higher risk for dyslipidemia yet without reaching statistical significance (OR = 1.06, 95% CI = 0.80–1.41) (Additional file 1: Table S2). However, individual parameter of dyslipidemia showed varying levels of risk associated with older age (Additional file 1: Table S3). Furthermore, there was a significant interaction between age and education level on dyslipidemia (*p* for interaction = 0.008), which was maintained in separate examination of hypercholesterolemia (*p* for interaction = 0.003) and hyper-LDL-cholesterolemia (*p* for interaction = 0.002) (Fig. 3). Sensitivity analyses based on secondary prevention levels indicated attenuated associations yet in parallel directions (Additional file 1: Table S4). Additionally, other ASCVD risk factors also modified age-cholesterol associations; there significant age by current smoking interactions on hypercholesterolemia (*p* for interaction = 0.018) and age by current drinking interactions on hypertriglyceridemia (*p* for interaction = 0.019) (Additional file 1: Table S3).

## Discussion

Our findings extend the conventional assay of dyslipidemia prevalence by illustrating the degree of effect modification that education level exerts on the association between age and dyslipidemia parameter, independent of other SES, behavioral, and reproductive risk factors. Overall, all dyslipidemia parameter worsened with older age, yet in non-linear manner. Furthermore, the degree of such deterioration differed by education level; notably, education level exerted a significant interaction on dyslipidemia, as a whole, and on its TC and LDLC components.

Such discrepancy is clinically concerning, especially in women, considering that there are substantial differences in CHD treatment and target lipid achievement by sex, age, and SES. A multinational study [21, 22] conducted by the European Society of Cardiology has reported worse cardiovascular risk profile in females across all age groups, with a significant sex by sex and education interaction [21]. Specifically, males were more likely non-obese, be equipped with smoking cessation aid, and to perform sufficient physical activity, thereby more likely to attain target LDLC and glycated hemoglobin levels. The subgroup analyses pinpointed the largest sex difference in less educated and elderly patients [22].Furthermore, this discriminative role of education in ASCVD risk factor control was more highlighted in women, where compliance with recommendations on lifestyle changes in patients with established CHD was inversely associated to SES in both primary and secondary prevention contexts [22].

Older age has historically been established as the most devastating contributor of dyslipidemia. Both cross-sectional and longitudinal studies have shown that TC, LDLC, and TG concentrations were positively associated with age, whilst a significant negative association with HDLC concentrations [23–26]. Such results were independent of ethnicity, race, and many other relevant risk factors. Our results also align with known lipid trajectories with biological aging; the Korean national data also showed that the mean levels of all serum lipid levels in women increased without upper threshold with aging, even exceeding that of men after sixth decade [27]. Hitherto, current treatment and surveillance guidelines highly advise precaution for elevated lipid levels among middle-aged and elderly women [19, 20, 28]. Furthermore, older adults were less likely to correctly recognize target blood pressure and cholesterol levels, suggesting insufficient awareness of cardiovascular risks for punctual management [29]. These findings highlight the need for improved promotion of ASCVD prevention in elderly population segment.

However, the role of education is still debated. In earlier Korean studies, only hypertriglyceridemia and hypoalphalipoproteinemia were inversely associated with education level in women [30]. Yet, a subsequent study demonstrated that all parameters of dyslipidemia are negatively associated with education level [13]. Inconsistencies are also observed in other nations’ studies. A Swiss study reported that lower levels of education were associated with high LDLC and TG levels in women [31]. South Asian studies showed that despite abnormal HDLC and LDLC were associated with increasing age, no concrete association was found with education level, occupation, and income category [26, 32]. Overview of multinational surveys conducted in clinic/population, urban/rural settings, low−/high-income, and middle-aged/elderly populations indicated wide-ranging (15–92%) hypoalphaliproteinemia prevalence [33]. In short, education level appears to assume multifaceted role in diverse ethnic, racial, and social frames.

The exact mechanisms underpinning the disproportionate role of education on dyslipidemia remain unclear. One possible explanation is that the interaction between unhealthy lifestyle and increased psychosocial stress activate inflammatory mediators, resulting in deleterious cardiovascular pathology [34]. People with low SES are more prone to unhealthy behaviors such as cigarette smoking, alcohol drinking, physical inactivity, and unbalanced diet [34, 35]. Although the prevalence of cigarette smoking is low in Korean population [36], relatively higher proportion of current smokers among our study participants in low education group may mediate the presence of significant interaction for hypercholesterolemia. Moreover, whereas higher education level is known to be associated with increased physical activity, high-caloric and low-nutrient food consumption is more frequently observed in women of lower level education [37, 38]. Such unfavorable health behaviors are known to induce psychosocial stress, thereby accelerating atherosclerotic process and succession. It has been suggested that persons of lower SES deploy less effective coping strategies and face more obstacles in accessing larger support networks, greater material resources, and healthcare to deal with stressful circumstances [39]. Moreover, slower recovery in cardiovascular responses after acute stress in persons of lower SES may contribute to atherosclerotic exacerbation [40]. Altogether, these can hinder appropriate lipid management. In sum, if unhealthy lifestyle attributable to low education level had contributed to each serum lipid differentially, such would explain significant age-education interaction observed only for high TC and LDLC levels in our findings.

Another explanation is given that different SES indicators operate in subtly different ways, their relationship with dyslipidemia may vary according to the index being used. A prospective Indian study showed that the prevalence of hypercholesterolemia and hypertriglyceridemia increased significantly in the lower income group, but observed no significant association with education level [41]. Therefore, different SES indicators may exert circumstantial effects in different populations.

Perhaps individual unit of SES is cannot adequately capture the risk associated with dyslipidemia. Ecologic studies have shown that people living in socioeconomically disadvantaged areas generally experience worse health outcomes than do those living in more affluent areas, independent of individual economic standing [42]. For instance, TG, but not HDLC, levels were higher in participants with greater neighborhood socioeconomic disadvantage than they were in those with less neighborhood socioeconomic disadvantage [43]. In a meta-analysis of African countries, the overall prevalence of elevated TC was determined by the geographic environment of residence, rather than individual education level [44]. Structural environment may more comprehensively capture the risk factors of dyslipidemia, such as deprivation, poorer access to health care, and lack of social support [45].

Divergent findings may reflect differences in methodological approaches. A longitudinal study that used inverse probability-weighted marginal structural model to estimate the controlled direct effect of adult SES on mortality, not mediated by health behaviors (accounting for potential confounding by time-varying health status), has confirmed the independent effect of SES [46]. Perhaps prospective study design examining the incidence, not prevalence, of dyslipidemia may better elucidate the true effect of education level. Additionally, simultaneous inclusion of multiple SES indicators in the same models estimating the effects of education generates an ambiguous causal parameter [46]. Statistical frameworks and adjustments may determine education effect calculation on clinical outcomes.

The strength of the study lies in its design and objective to collect diverse and in-depth information on traditional and emerging risk factors and biomarkers of cardiometabolic disorders, which manifests through adverse lipid profile. Moreover, the study population embodies diverse SES and physiological background of community-dwelling, middle-aged women, thereby strengthening external validity to the general Korean population. Many covariates, especially reproductive history, which is regarded as an independent risk factor of dyslipidemia, was measured at extensive depth and detail. Many studies have demonstrated profound effect of menopause and hormone-regulating medications on lipid concentrations [47, 48] our results remained robust after adjusting for numerous possible pathways that may otherwise distort the association. Lastly, our results remained consistent even after substituting the current primary prevention criteria with more conservative treatment goal cutoff values recommended by multinational guidelines. This may extend the modifiability of education level on the association between age and lipid levels, even at subclinical levels.

However, there are limitations to be considered. First, the cross-sectional nature of our study only enabled us to consider SES indicators and health behaviors as time-fixed variables. However, adjusting for these covariates measured at one point in time (in our case, adulthood) may distort the lifetime contribution of education level to dyslipidemia. Study has shown that the early-life SES also have independent or mediating effects on adult-onset of chronic diseases and mortality [49]. Another concern is the absence of information regarding predisposed and non-modifiable risk factors. There is a growing body of evidence that combinations of multiple genes harboring predisposing alleles have causal role to the population variance of lipid levels [50–52]. Combined with the single nucleotide polymorphisms, behavioral-environmental interactions affect cholesterols [2]. However, our study lacked information on familial lipid abnormality nor population-specific SNP assay; thus, hereditary information could not be adjusted for. Furthermore, because single-occasion serum lipid measurements were used to classify dyslipidemia, measurement variability cannot be ruled out. Lastly, because demographic information and the use of lipid-lowering medications were obtained via self-report, response bias cannot be ruled out.

## Conclusion

The current study investigated the differential role of education level on the association between age and individual parameter of dyslipidemia in community-dwelling, middle-aged women. While education level is difficult to modify in middle-aged population, tailored lifestyle modification education programs can be implemented to raise dyslipidemia awareness and control across all age groups. Considering that elevated lipid levels are highly reversible even with sustained healthy lifestyle, timely intervention may deter the atherosclerotic process, thereby conserving substantial healthcare burden at both individual and national level. Meanwhile, future studies are warranted to further elucidate the role of education level on lipid profiles even across young and elderly populations, whom we expect heterogeneous educational background. Alternative features, such as the length of exposure to dyslipidemia, genetic and biological susceptibility, or lipoprotein subfractions, may supplement our current understandings on this complex interplay.

## Supplementary information

**Additional file 1 Figure S1**. Flow chart of the selection criteria for the final study population (*n* = 2049). **Table S1.** Distribution of lipid levels by age group and education level (*n* = 2049). **Table S2.** Association between age and dyslipidemia prevalence according to education level and other known risk factors using a generalized linear model (*n* = 2049). **Table S3.** Association between age and individual dyslipidemia parameters according to education level and other known risk factors using a generalized linear model (*n* = 2049). **Table S4.** Association between age and LDL cholesterol by the secondary prevention target goals according to education level and other known risk factors using a generalized linear model (*n* = 2049).

## Data Availability

We have uploaded data for each survey year to iCReaT, the clinical research information management system of the Korea National Institute of Health. We also keep biospecimens such as serum, plasma, buffy coat, and urine for future use, after obtaining individual consent for the retention period and scope of use. Biospecimens will be deposited at the Korea Biobank, managed by the Korea Centers for Disease Control and Prevention, after completion of the baseline assessment. Although cohort enrollment and baseline assessment are ongoing, this study is open to interested researchers. Researchers interested in collaborative study are invited to contact the CMERC principal investigator, Hyeon Chang Kim, at hckim@yuhs.ac.

## Abbreviations

ASCVD: Atherosclerotic cardiovascular disease
CHD: Coronary heart disease
CMERC: Cardiovascular and metabolic disease etiology research center
HDLC: High-density lipoprotein cholesterol
LDLC: Low-density lipoprotein cholesterol
OR: Odds ratio
SES: Socioeconomic status
TC: Total cholesterol
TG: Triglyceride
