# Supplementary material for: Associations between age and dyslipidemia are differed by education level: The Cardiovascular and Metabolic Diseases Etiology Research Center (CMERC) cohort
Source: Lipids Health Dis. 2020 Jan 18;19:12. doi: 10.1186/s12944-020-1189-y (PMC6969451; doi:10.1186/s12944-020-1189-y)
Supplement: Supplementary file 1 — Additional file 1 Figure S1. Flow chart of the selection criteria for the final study population (n = 2049). Table S1. Distribution of lipid levels by age group and education level (n = 2049). Table S2. Association between age and dyslipidemia prevalence according to education level and other known risk factors using a generalized linear model (n = 2049). Table S3. Association between age and individual dyslipidemia parameters according to education level and other known risk factors using a generalized linear model (n = 2049). Table S4. Association between age and LDL cholesterol by the secondary prevention target goals according to education level and other known risk factors using a generalized linear model (n = 2049). [file 12944_2020_1189_MOESM1_ESM.docx]

**Supplementary Figure 1**. Flow chart of the selection criteria for the final study population (n=2,049).


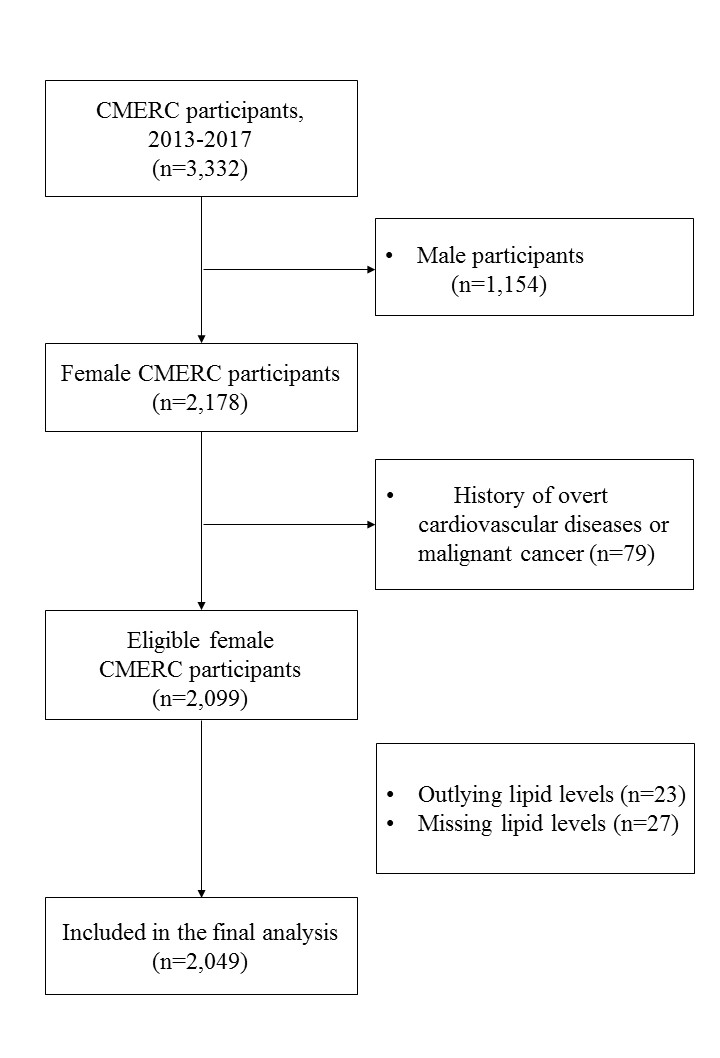


**Supplementary Table 1.** Distribution of lipid levels by age group and education level (n=2,049).

| **Supplementary Table 1.** Distribution of lipid levels by age group and education level (n=2,049). | | | | | | | | | | | | | | |
| --- | --- | --- | --- | --- | --- | --- | --- | --- | --- | --- | --- | --- | --- | --- |
| Lipid variable | Age, year | | | | | | | | | | | |  | *p*-value☨ |
|  | 30-39 | | | 40-49 | | | 50-59 | | | 60-64 | | |  |  |
| **Low education level** |  |  |  |  |  |  |  |  |  |  |  |  |  |  |
| Total cholesterol | 183.3 | ± | 28.7 | 196.3 | ± | 31.1 | 204.2 | ± | 34.5 | 199.5 | ± | 31.1 |  | <0.0001 |
| Triglyceride, logarithmic | 4.4 | ± | 0.5 | 4.5 | ± | 0.4 | 4.7 | ± | 0.4 | 4.8 | ± | 0.4 |  | <0.0001 |
| HDL cholesterol | 63.5 | ± | 15.8 | 63.9 | ± | 14.2 | 60.1 | ± | 14.3 | 58.1 | ± | 13.8 |  | <0.0001 |
| LDL cholesterol | 100.5 | ± | 27.8 | 111.9 | ± | 28.2 | 120.3 | ± | 30.3 | 116 | ± | 29.0 |  | <0.0001 |
| **High education level** |  |  |  |  |  |  |  |  |  |  |  |  |  |  |
| Total cholesterol | 180.5 | ± | 27.9 | 194.6 | ± | 30.1 | 208.9 | ± | 32.9 | 204.5 | ± | 37.8 |  | <0.0001 |
| Triglyceride, logarithmic | 4.3 | ± | 0.4 | 4.5 | ± | 0.4 | 4.6 | ± | 0.4 | 4.8 | ± | 0.4 |  | <0.0001 |
| HDL cholesterol | 62.6 | ± | 14.0 | 61.7 | ± | 14.2 | 62.7 | ± | 14.6 | 58.3 | ± | 12.5 |  | 0.0531 |
| LDL cholesterol | 101.8 | ± | 23.8 | 113.6 | ± | 25.3 | 123.8 | ± | 28.9 | 120.6 | ± | 35.5 |  | <0.0001 |
| The unit is in mg/dL, except for logarithmic conversion of triglyceride. | | | | | | | | | | | | | | |
| Values are presented as mean ± standard deviation. | | | | | | | | | | | | | | |
| ^☨^*P-*value was derived from the analysis of variance test for multiple comparisons. | | | | | | | | | | | | | | |
| Low education level refers to completion of high school or below; high education level refers to completion of college or above. | | | | | | | | | | | | | | |
| Abbreviation: HDL, high-density lipoprotein; LDL, low-density lipoprotein | | | | | | | | | | | | | | |

**Supplementary Table 2.** Association between age and dyslipidemia prevalence according to education level and other known risk factors using a generalized linear model (n=2,049).

| **Supplementary Table 2.** Association between age and dyslipidemia prevalence according to education level and | | | | | |
| --- | --- | --- | --- | --- | --- |
| other known risk factors using a generalized linear model (n=2,049). | | | | | |
| Subgroup | No. | No. | OR (95% CI)* | *p-*value | *p* for interaction☨ |
|  | participants | case |  |  |  |
| Total participants | 2,049 | 821 | 1.06 (0.80-1.41) | 0.69 |  |
|  |  |  |  |  |  |
| Education level |  |  |  |  |  |
| Low | 1,245 | 577 | 1.58 (1.19-2.10) | 0.01 | 0.008 |
| High | 804 | 244 | 2.31 (1.58-3.40) | <0.0001 |  |
| Menopause |  |  |  |  |  |
| No | 762 | 157 | 0.28 (0.03-2.51) | 0.26 | 0.741 |
| Yes | 1,287 | 664 | 1.10 (0.82-1.46) | 0.53 |  |
| Obese, BMI ≥25 kg/m² |  |  |  |  |  |
| No | 1,508 | 510 | 1.98 (1.50-2.61) | <0.0001 | 0.049 |
| Yes | 541 | 311 | 1.50 (1.01-2.25) | 0.05 |  |
| Current drinking |  |  |  |  |  |
| No | 761 | 332 | 1.65 (1.14-2.39) | 0.01 | 0.118 |
| Yes | 1,288 | 489 | 1.98 (1.49-2.65) | <0.0001 |  |
| Current smoking |  |  |  |  |  |
| No | 1,985 | 790 | 1.85 (1.47-2.33) | <0.0001 | 0.208 |
| Yes | 64 | 31 | 0.92 (0.16-5.43) | 0.92 |  |
| ^*^The model is adjusted for body mass index, reproductive history, household income, occupation, education level, | | | | | |
| alcohol consumption, cigarette smoking, physical activity, alcohol intake, and lipid-lowering agent. | | | | | |
| Low education level refers to completion of high school or below; high education level refers to completion of | | | | | |
| college or above. | | | | | |
| ☨The *p-*values for interactions between age and known risk factors were calculated from Wald statistics. | | | | | |
| Abbreviation: BMI, body mass index; CI, confidence interval; OR, odds ratio | | | | | |

**Supplementary Table 3.** Association between age and individual dyslipidemia parameters according to education level and other known risk factors using a generalized linear model (n=2,049).

| Parameters | Subgroup | No. | No. | OR (95% CI)* | *p-*value | *p* for interaction☨ |
| --- | --- | --- | --- | --- | --- | --- |
|  |  | participants | case |  |  |  |
| Hypercholesterolemia | Total participants | 2,049 | 246 | 0.85 (0.60-1.19) | 0.33 |  |
|  |  |  |  |  |  |  |
|  | Education level |  |  |  |  |  |
|  | Low | 1,245 | 162 | 1.30 (0.90-1.88) | 0.16 | 0.003 |
|  | High | 804 | 84 | 2.61 (1.58-4.29) | 0.0002 |  |
|  | Menopause |  |  |  |  |  |
|  | No | 762 | 46 | 0.85 (0.09-8.21) | 0.89 | 0.488 |
|  | Yes | 1,287 | 200 | 0.81 (0.58-1.15) | 0.24 |  |
|  | Obese, BMI ≥25 kg/m² |  |  |  |  |  |
|  | No | 1,508 | 151 | 1.88 (1.28-2.76) | 0.001 | 0.315 |
|  | Yes | 541 | 95 | 1.32 (0.81-2.16) | 0.27 |  |
|  | Current drinking |  |  |  |  |  |
|  | No | 761 | 88 | 1.33 (0.80-2.20) | 0.27 | 0.193 |
|  | Yes | 1,288 | 158 | 1.97 (1.35-2.87) | 0.0004 |  |
|  | Current smoking |  |  |  |  |  |
|  | No | 1,985 | 238 | 1.80 (1.32-2.44) | 0.0002 | 0.018 |
|  | Yes | 64 | 8 | 1.01 (0.29-N/A) | 0.95 |  |
| Hypertriglyceridemia | Total participants | 2,049 | 365 | 0.99 (0.72-1.36) | 0.93 |  |
|  |  |  |  |  |  |  |
|  | Education level |  |  |  |  |  |
|  | Low | 1,245 | 255 | 1.44 (1.04-2.00) | 0.03 | 0.461 |
|  | High | 804 | 110 | 1.45 (0.90-2.32) | 0.13 |  |
|  | Menopause |  |  |  |  |  |
|  | No | 762 | 79 | 1.01 (0.22-N/A) | 0.98 | 0.297 |
|  | Yes | 1,287 | 286 | 1.01 (0.73-1.40) | 0.94 |  |
|  | Obese, BMI ≥25 kg/m² |  |  |  |  |  |
|  | No | 1,508 | 207 | 1.45 (1.02-2.05) | 0.04 | 0.548 |
|  | Yes | 541 | 158 | 1.39 (0.91-2.12) | 0.12 |  |
|  | Current drinking |  |  |  |  |  |
|  | No | 761 | 134 | 1.23 (0.80-1.90) | 0.35 | 0.019 |
|  | Yes | 1,288 | 231 | 1.73 (1.24-2.43) | 0.002 |  |
|  | Current smoking |  |  |  |  |  |
|  | No | 1,985 | 350 | 1.51 (1.15-1.98) | 0.003 | 0.522 |
|  | Yes | 64 | 15 | 0.87 (0.09-8.22) | 0.91 |  |
| Hypoalphalipoproteinemia | Total participants | 2,049 | 84 | 1.17 (0.61-2.27) | 0.64 |  |
|  |  |  |  |  |  |  |
|  | Education level |  |  |  |  |  |
|  | Low | 1,245 | 59 | 1.01 (0.55-1.85) | 0.98 | 0.463 |
|  | High | 804 | 25 | 1.34 (0.55-3.31) | 0.52 |  |
|  | Menopause |  |  |  |  |  |
|  | No | 762 | 24 | 1.00 (0.03-N/A) | 0.99 | 0.429 |
|  | Yes | 1,287 | 60 | 1.33 (0.67-2.64) | 0.42 |  |
|  | Obese, BMI ≥25 kg/m² |  |  |  |  |  |
|  | No | 1,508 | 53 | 0.73 (0.38-1.39) | 0.34 | 0.187 |
|  | Yes | 541 | 31 | 2.14 (0.89-5.11) | 0.09 |  |
|  | Current drinking |  |  |  |  |  |
|  | No | 761 | 40 | 1.40 (0.66-2.97) | 0.38 | 0.916 |
|  | Yes | 1,288 | 44 | 1.01 (0.51-2.00) | 0.99 |  |
|  | Current smoking |  |  |  |  |  |
|  | No | 1,985 | 78 | 1.17 (0.70-1.97) | 0.54 | 0.693 |
|  | Yes | 64 | 6 | 1.78 (0.04-75.89) | 0.76 |  |
| Hyper-LDL-cholesterolemia | Total participants | 2,049 | 160 | 0.89 (0.59-1.35) | 0.58 |  |
|  |  |  |  |  |  |  |
|  | Education level |  |  |  |  |  |
|  | Low | 1,245 | 101 | 1.07 (0.69-1.68) | 0.76 | 0.002 |
|  | High | 804 | 59 | 2.85 (1.59-5.09) | 0.0004 |  |
|  | Menopause |  |  |  |  |  |
|  | No | 762 | 33 | 1.00 (0.09-N/A) | 0.98 | 0.780 |
|  | Yes | 1,287 | 127 | 0.91 (0.60-1.39) | 0.67 |  |
|  | Obese, BMI ≥25 kg/m² |  |  |  |  |  |
|  | No | 1,508 | 96 | 1.89 (1.18-3.02) | 0.01 | 0.069 |
|  | Yes | 541 | 64 | 1.00 (0.56-1.77) | 0.99 |  |
|  | Current drinking |  |  |  |  |  |
|  | No | 761 | 64 | 1.11 (0.63-1.97) | 0.72 | 0.096 |
|  | Yes | 1,288 | 96 | 1.97 (1.24-3.14) | 0.004 |  |
|  | Current smoking |  |  |  |  |  |
|  | No | 1,985 | 156 | 1.61 (1.12-2.32) | 0.01 | 0.148 |
|  | Yes | 64 | 4 | 1.04 (0.19-N/A) | 0.91 |  |
| ^*^The model is adjusted for body mass index, reproductive history, household income, occupation, education level, alcohol consumption, | | | | | | |
| cigarette smoking, physical activity, alcohol intake, and lipid-lowering agent. | | | | | | |
| Low education level refers to completion of high school or below; high education level refers to completion of college or above. | | | | | | |
| ☨The *p*-values for interactions between age and known risk factors were calculated from Wald statistics. | | | | | | |
| Abbreviation: BMI, body mass index; CI, confidence interval; LDL, low-density lipoprotein; OR, odds ratio | | | | | | |

**Supplementary Table 4.** Association between age and LDL cholesterol by the secondary prevention target goals according to education level and other known risk factors using a generalized linear model (n=2,049).

| **Supplementary Table 4.** Association between age and LDL cholesterol by the secondary prevention target goals according to | | | | | | |
| --- | --- | --- | --- | --- | --- | --- |
| education level and other known risk factors using a generalized linear model (n=2,049). | | | | | | |
|  | Subgroup | No. | No. | OR (95% CI)* | *p-*value | *p* for interaction☨ |
|  |  | participants | case |  |  |  |
| LDL cholesterol ≥130 mg/dL | Total participants | 2,048 | 620 | 0.92 (0.78-1.28) | 0.65 |  |
|  |  |  |  |  |  |  |
|  | Education level |  |  |  |  |  |
|  | Low | 1,245 | 389 | 1.11 (0.77-1.49) | 0.55 | 0.009 |
|  | High | 804 | 231 | 2.36 (1.42-3.19) | 0.01 |  |
|  | Menopause |  |  |  |  |  |
|  | No | 762 | 109 | 1.04 (0.75-1.41) | 0.95 | 0.810 |
|  | Yes | 1,287 | 511 | 0.93 (0.74-1.28) | 0.67 |  |
|  | Obese, BMI ≥25 kg/m² |  |  |  |  |  |
|  | No | 1,508 | 361 | 1.63 (1.20-2.88) | 0.01 | 0.053 |
|  | Yes | 541 | 259 | 1.11 (0.60-1.65) | 0.89 |  |
|  | Current drinking |  |  |  |  |  |
|  | No | 761 | 271 | 1.08 (0.60-1.74) | 0.84 | 0.069 |
|  | Yes | 1,288 | 349 | 1.60 (1.17-2.97) | 0.01 |  |
|  | Current smoking |  |  |  |  |  |
|  | No | 1,985 | 590 | 1.61 (1.12-2.32) | 0.01 | 0.081 |
|  | Yes | 64 | 30 | 1.70 (0.94-3.20) | 0.21 |  |
| LDL cholesterol ≥116 mg/dL | Total participants | 2,048 | 990 | 0.96 (0.69-1.34) | 0.78 |  |
|  |  |  |  |  |  |  |
|  | Education level |  |  |  |  |  |
|  | Low | 1,245 | 609 | 1.08 (0.79-1.43) | 0.63 | 0.013 |
|  | High | 804 | 381 | 1.77 (1.19-2.99) | 0.01 |  |
|  | Menopause |  |  |  |  |  |
|  | No | 762 | 269 | 0.97 (0.66-1.32) | 0.96 | 0.921 |
|  | Yes | 1,287 | 721 | 1.01 (0.77-1.28) | 0.94 |  |
|  | Obese, BMI ≥25 kg/m² |  |  |  |  |  |
|  | No | 1,508 | 589 | 1.41 (1.09-2.14) | 0.03 | 0.062 |
|  | Yes | 541 | 401 | 1.14 (0.65-1.72) | 0.90 |  |
|  | Current drinking |  |  |  |  |  |
|  | No | 761 | 446 | 0.98 (0.68-1.66) | 0.93 | 0.201 |
|  | Yes | 1,288 | 544 | 1.24 (0.95-2.71) | 0.11 |  |
|  | Current smoking |  |  |  |  |  |
|  | No | 1,985 | 946 | 1.35 (0.94-1.89) | 0.09 | 0.105 |
|  | Yes | 64 | 44 | 1.55 (0.91-2.97) | 0.23 |  |
| ^*^The model is adjusted for body mass index, reproductive history, household income, occupation, education level, alcohol consumption, | | | | | | |
| cigarette smoking, physical activity, alcohol intake, and lipid-lowering agent. | | | | | | |
| Low education level refers to completion of high school or below; high education level refers to completion of college or above. | | | | | | |
| ☨The *p*-values for interactions between age and known risk factors were calculated from Wald statistics. | | | | | | |
| Abbreviation: BMI, body mass index; CI, confidence interval; LDL, low-density lipoprotein; OR, odds ratio | | | | | | |
